# Effects of frequency and amount of stover mulching on the microbial community composition and structure in the endosphere and rhizosphere

**DOI:** 10.3389/fmicb.2024.1372471

**Published:** 2024-02-26

**Authors:** Haowen Li, Yawen Zou, Wenchen Song, Jiaxun Xin, Jian Gao

**Affiliations:** ^1^College of Life and Environmental Sciences, Minzu University of China, Beijing, China; ^2^School of Ecology and Environment, Baotou Teachers’ College, Inner Mongolia University of Science and Technology, Baotou, China

**Keywords:** frequency of stover mulching, amount of stover mulching, microbial community, endosphere, rhizosphere

## Abstract

Stover mulching, as a sustainable agricultural conservation practice, has been shown to effectively increase soil organic matter and enhance crop yields. The impact of stover mulching on soil microorganisms has been extensively studied. However, less attention has been given to endophytic and rhizospheric microorganisms that have closer relationships with crops. How do the quality and frequency of stover mulching affect the composition and structure of these endosphere and rhizosphere microbial communities? And what is their influence on critical indicators of soil health such as bacterial plant pathogen and Rhizobiales? These questions remain unresolved. Therefore, we investigated the responses of the microbial functional guilds in the endosphere and rhizosphere to maize stover mulching qualities (0%, 33%, 67%, and total stover mulching every year) and frequencies (once every 3 years and twice every 3 years) under 10-year no-till management. Results showed significant correlations between Bacillales and Rhizobiales orders and soil SOC, NO_3_^−^N, and NH_4_^+^N; Hypocreales and Eurotiales orders were significantly correlated with soil NO_3_^−^N, with the Aspergillus genus also showing a significant correlation with soil SOC. The frequency and quality of stover mulching had a significant effect on root and rhizospheric microbial communities, with the lowest relative abundance of bacterial plant pathogens and highest relative abundance of nitrogen-fixing bacteria such as Rhizobiales and Hypocreales observed under F1/3 and F2/3 conditions. The most complex structures in endosphere and rhizospheric microbial communities were found under Q33 and Q67 conditions, respectively. This research indicates that from a soil health perspective, low-frequency high-coverage stover mulching is beneficial for the composition of endosphere and rhizosphere microbial communities, while moderate coverage levels are conducive to more complex structures within these communities. This study holds significant ecological implications for agricultural production and crop protection.

## 1 Introduction

Maize (*Zea mays* L.) is one of the world’s three principal cereal crops. As an agricultural powerhouse, China contributes to 23% of the global maize production (Wang Y. Q. et al., 2019). However, this substantial yield also generates a considerable amount of crop residue, particularly cornstalks, which require proper disposal. In the past, many farmers opted for field burning due to the high costs associated with managing these residues. This practice led to the emission of particulate matter, nitrogen oxides, sulfur dioxide, and other pollutants into the atmosphere, causing air pollution, nutrient loss, and posing health risks to humans (Wang Z. Z. et al., 2019; Cao et al., 2022). In line with sustainable agricultural development in China, it is imperative to utilize crop residue resources effectively. Stover mulching, as a key method for such utilization, has been shown to increase soil organic matter content and enhance crop yields, thus contributing positively to agricultural sustainability (Liu et al., 2010).

In soil, there exists a diverse community of microorganisms that play crucial roles in energy flow, nutrient cycling, and information exchange (Chaparro et al., 2012; Frąc et al., 2018), serving as biological indicators of soil health (Doran and Zeiss, 2000). A wealth of research has demonstrated significant differences in the microbial community composition between soils subjected to stover mulching compared to those under conventional tillage practices (Zhang et al., 2019; Yang et al., 2022). Overall, stover mulching tends to shift soil microbial communities toward a composition that is more conducive to crop growth; however, it can also introduce potential drawbacks, such as an increase in pests and diseases (Gao et al., 2022). Studies have shown that both the quantity and frequency of stover mulching directly impact the soil microbial community, with specific frequencies and quantities capable of effectively steering the microbial populations toward a composition that benefits crops (Wang et al., 2020a; Yang et al., 2022). Therefore, altering the quantity and frequency of stover mulching may be a critical strategy for optimizing this practice while minimizing potential risks.

The rhizosphere, the narrow region surrounding plant roots within a few millimeters, is a critical zone where microorganisms engage in intensive material exchange and information transfer with plants, often hosting the most active soil microbial communities (Hassan et al., 2019; Song et al., 2023). The endosphere refers to the internal plant tissues where diverse microorganisms coexist symbiotically within the plant (Adeleke et al., 2023), and endophytic microorganisms are more similar in the ecological niche to “producers” (Song et al., 2023). Compared to soil microorganisms, both endophytes and rhizosphere microbes have more direct interactions with plants, being more intimately related to plant health and functioning (Attia et al., 2021). In evaluating the impact of stover mulching on soil health, investigating changes in the endophytic and rhizosphere microbial communities are of paramount importance. However, there is currently a dearth of research specifically addressing how stover mulching affects endophytes and rhizosphere microbes within the root system. It remains uncertain whether the effects of stover mulching on endophytes and rhizosphere microbiota are merely the extension of its influence on the soil microbial community or if they involve more intricate and unique relationships. Existing studies have indeed assessed the effects of variations in stover mulching frequency and amount on nitrifying bacterial and fungal plant pathogens within both the endosphere and rhizosphere (Song et al., 2022), with some research delving down to the species level (Wang et al., 2020a,b). However, there remain substantial gaps in the research on how the quality and frequency of stover mulching influence endosphere and rhizosphere microbial communities, particularly with respect to critical groups such as rhizobia and bacterial plant pathogens.

Stover mulching is a process involving the release and mineralization of organic nutrients, with numerous factors influencing its decomposition, including soil properties, types of microorganisms, and hydrothermal conditions. Microorganisms are core elements in the cycling and transformation of soil carbon and nitrogen, and primarily rely on these organisms to decompose stover and release nutrients into the soil (Hu et al., 2012). Typically, the decomposition of initial components such as proteins and cellulose in straw is predominantly carried out by bacteria (Marschner et al., 2011), while fungi play a major role in the breakdown of more recalcitrant components like lignin during later stages. However, existing studies have shown that bacteria play a significant role throughout the entire process of straw decomposition (Lee et al., 2011; Fan et al., 2014a). Throughout the process of stover decomposition, microorganisms constantly interact with soil and plants. Their biological activities have a direct or indirect impact on soil physical properties as well as plant health and productivity (Xiong et al., 2021; Zhu et al., 2021). Research has shown that the composition and functions of endophytic and rhizospheric microbiota significantly vary across different growth stages of plants, exerting distinct ecological roles (Xiong et al., 2020, 2021). Beyond the interactions between environmental factors and microbial communities, there are also complex interrelationships among different microorganisms within these communities. Prior study have demonstrated that stover mulching can increase the complexity of microbial interaction networks (Wang et al., 2020b). Thus, it is hypothesized that the quality and frequency of stover mulching could affect the connectivity between endophytic and rhizospheric microbial populations, with varying degrees of influence. However, current research largely focuses on the impacts of mulch quantity and frequency on overall soil microbial communities (Wang et al., 2021), with relatively little attention given to their specific effects on endophytic and rhizospheric microbiota. Currently, there is a substantial body of research examining the effects of stover mulching quantity and frequency on soil microbial communities across various crops such as rice, wheat, soybeans, among others (Wang et al., 2021, 2023; Wei et al., 2021), including corn which is under investigation in our study. However, literature addressing the specific impacts of straw return on endophytic and rhizosphere microbial populations is scarce. As a result, the precise influence of different stover mulch frequencies and amounts on the composition and structure of both the endosphere and rhizosphere microbial communities remains largely unknown.

Previous studies on soil microbial communities showed that a high-frequency while small-quantity mulch is more beneficial for soil microbial communities (Kou et al., 2020; Yang et al., 2022). Thus, our research hypothesis is that this phenomenon will continue to endophytic and rhizosphere microbial communities, where a high-frequency while small-quantity mulch is also beneficial to endophytic and rhizosphere microbial communities. This study aims to investigate the effects of different stover mulching frequencies and qualities on the composition and structure of endophytic and rhizospheric microbial communities at the order and lifestyle levels. The objective is to identify optimal frequencies and quantities of straw return from a perspective of maintaining and promoting the health of microbial communities, which holds significant ecological implications for the protection and enhancement of agricultural production.

## 2 Materials and methods

### 2.1 Experimental design

Field trials were conducted in the spring of 2010 in a maize cropping system in Aohan, Inner Mongolia (42.26′N, 119.70′E). Maize stover was used in the field trials. The local average annual temperature is 6°C and the average annual precipitation is 384 mm, with more than 70% of the total precipitation falling between June and September. It has a monsoon, continental and semi-arid climate, and the soil is yellow-brown clay loam.

The land was plowed for 10 years. The trial has conducted over 9 years with six randomized treatments (Supplementary Figure S1): Q0 (no return control), Q33 (33% stover mulched per year), Q67 (67% stover mulched per year), Q100 (100% stover mulched per year), F1/3 (stover mulched in the first year of the three-year cycle) and F2/3 (stover mulched in the first 2 years of the three-year cycle). Compound fertilizer (N-P_2_O_5_-K_2_O, 26%–12%) was applied at a rate of 900 kg/ha before maize was sown. After maize was harvested, plant parts other than residual stubble (about 30 cm high) were crushed and then mulched to the soil surface according to the proportion of the experimental treatments, with a maximum application rate (Q100) of 7.5 t/ha.

### 2.2 Field sampling and laboratory analysis

The soil was sampled continuously in 2020 at the time of jointing (May to June), flowering (July) and mature (September) stages of maize, and the roots of five plants were randomly collected as samples in each plot. After removing dead leaves, stones and other impurities, in order to isolate the rhizosphere soil, fine roots less than 2 mm in diameter were packed into 2 mL sterile tubes with sterile water, shaken for 15 min then centrifuged at 10,000 r/min. At the end of centrifugation, the fine roots were rinsed more than 3 times with deionized sterile water to ensure that all soil on the root surface was washed, and then stored at −80°C together with the rhizosphere soil obtained by centrifugation. Soil for chemical analyses was sampled instead by using an auger to collect undisturbed bulk soil, sieved through a 2 mm sieve and stored at 4°C. The soil was analyzed using the method described by Kou et al. (2020) for the determination of total soil nitrogen (TN), nitrate nitrogen (NO_3_^−^N) and ammonium nitrogen (NH_4_^+^N), and the wet oxidation method was used for the determination of soil organic carbon (SOC).

### 2.3 DNA extraction

DNA extraction was performed using the method of Song et al. (2020). Maize roots were to be pre-treated, submerged in liquid nitrogen, and then MO BIO’s PowerSoil DNA isolation kit (Qiagen, Germany)was used to extracted DNA (0.5 g) from rhizosphere soil and root samples, following the manufacturer’s instructions. The concentration of extracted DNA was determined using a NanoDrop 2000 spectrophotometer (Waltham, Massachusetts, United States). To study the bacterial and fungal communities, targeted amplification of the V4 region of bacterial 16S rRNA and the ITS2 region of fungal ITS DNA, respectively, was performed with the universal primers 515F (GTGCCAGCMGCCGCGGGTAA) and 806R (GGACTACHVGGGGTWTCTAAT; for bacterial 16SrRNA) and 5.8SFun (AACTTTYRRCAAYGGGATCWCT) and ITS4Fun (AGCCTCCGCTTATTGATATGCTTAART; for fungal ITS regions). A mixture consisting of 1 μL DNA, 2.5 μL forward and reverse primers, and 5 μL PCR buffer was added to the PCR system. The PCR procedure consisted of a 3-min maintenance at 95°C (denaturation); then three stages of 30-s maintenance at 95°C, 30-s maintenance at 55°C, and 45-s maintenance at 72°C in one cycle, which was repeated 27 times; and a final extension of 72°C for 10 min. The PCR reaction process and product purification were performed according to the method described by Taş et al. (2014). Amplicon libraries were sequenced using the Illumina MiSeq platform (Illumina, United States) with a paired-end sequencing strategy.

### 2.4 Bioinformatic analysis

Valid sequences were first filtered from the raw data; sequences that were too short in length (<230 bp), had low quality scores (≤20), contained indeterminate bases, or primer sequences that did not exactly match the barcode tag were disregarded and removed with sample-specific barcode sequences. Sequences obtained from the sequencer were processed using the QIIME2 pipeline (Caporaso et al., 2010). After removal of barcodes and primers, ambiguous reads and low quality sequences were removed to improve sequence quality. Paired ends of 16S rRNA and ITS reads were merged using the FLASH tool (Magoc and Salzberg, 2011). Chimeric sequences were removed and valid sequences with more than 97% similarity were grouped into operational taxonomic units (OTUs) using UPARSE. Representative sequences for each bacterial and fungal OTU were searched for taxonomic similarity using the SILVA 138 SSU Ref NR99 and UNITE 8.2 databases (Nilsson et al., 2018). The entire database has been submitted to the NCBI Sequence Read Archive (SRA) database under accession number PRJNA758631.

### 2.5 Statistical analyses

Statistical analyses were performed using the R (4.0.2) platform and data visualization was performed using the R package “ggplot2” (3.3.6). The aov() function and corr.test() function in the statistical package (version 4.0.2) were used to perform ANOVA to assess the significance of microbial abundance among treatments and Spearman’s correlation analysis to predict the relationship between microbial communities and soil chemical properties, respectively. Species composition was analyzed by histograms using the R software packages psych (2.2.5), and correlation heatmaps were generated using pheatmap (1.0),. Tukey’s HSD test was used if differences between groups were significant. Bacterial and fungal functions were delineated separately using FAPROTAX and FungalTraits (Louca et al., 2016; Põlme et al., 2020). Symbiotic patterns of bacterial and fungal communities were assessed through network analyses using maximum information coefficient (MIC) scores from the MINE statistic (Shannon et al., 2003). The networks were then visualized in Cytoscape version 3.4.0 (Reshef et al., 2011). The NetworkAnalyzer tool was used to calculate network topology parameters (see Romdhane et al., 2022 for details).

## 3 Results

### 3.1 Effects of stover mulching quantity and frequency on soil microbial lifestyle

**Figure 1:**
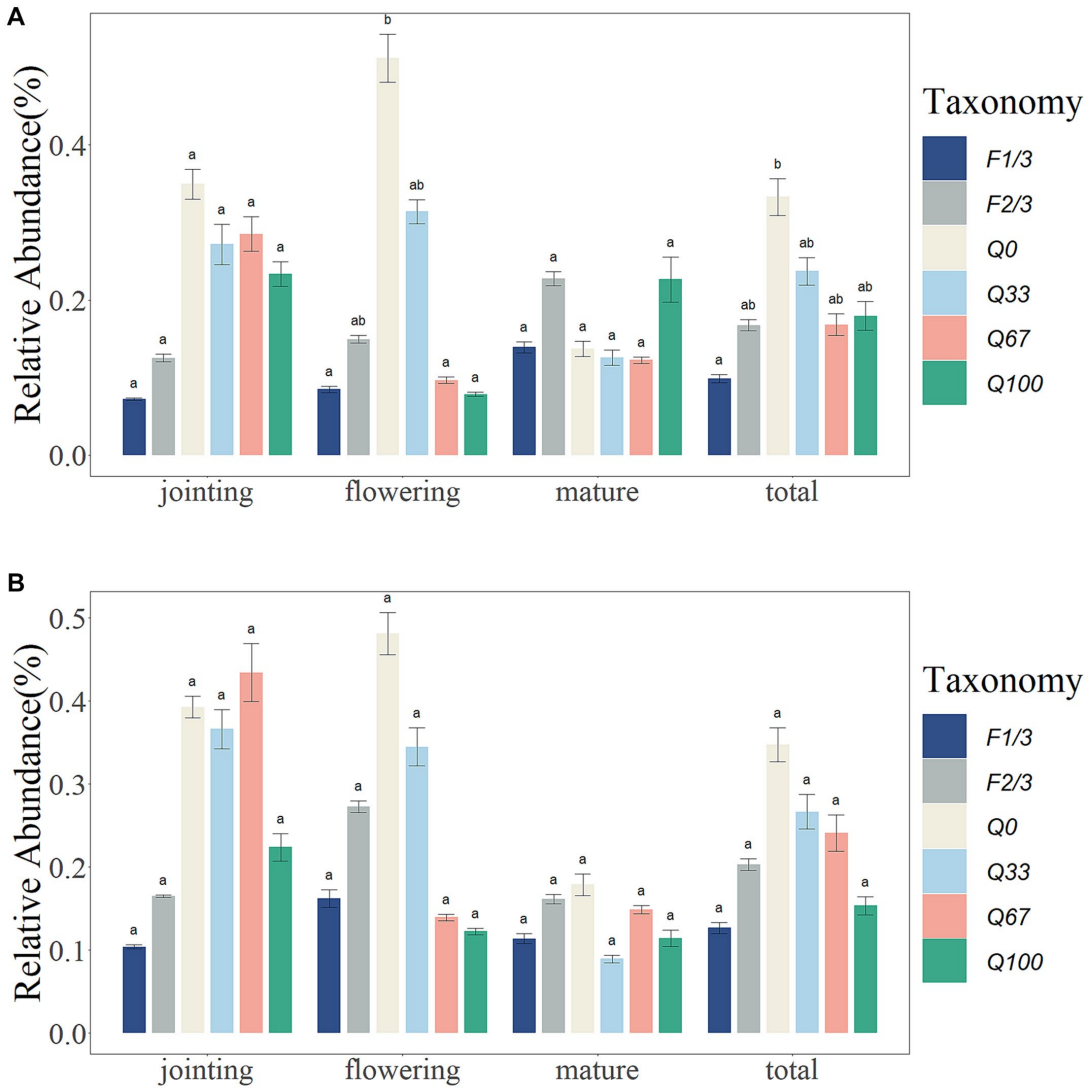
Relative abundance of bacterial plant pathogen in the endosphere **(A)** and rhizosphere **(B)** at different time periods and at different quantities and frequencies of stover mulching. Different letter means a statistical significance: *p* < 0.05.

Overall, different stover mulching treatments showed a suppressive effect on the relative abundance of bacterial plant pathogens, particularly within the endosphere. F1/3 had significantly lower relative abundances of bacterial plant pathogens compared Q0 in the endosphere (Figure 1A). During the jointing stage, the relative abundance of bacterial plant pathogens was significantly lower in the endosphere under the F1/3 and F2/3 treatments, while within the rhizosphere, there was a comparably lower relative abundance of bacterial plant pathogens observed under the F1/3, F2/3, and Q100 treatment regimens. At the flowering stage, the relative abundances of plant pathogens in the endosphere were significantly lower in the F1/3, Q67, and Q100 than in the Q0, with all experimental groups showing decreased relative abundances of bacterial plant pathogens in both the endosphere and rhizosphere as compared to Q0 (Figure 1A). However, during the mature stage, the impact of various stover mulching qualities and frequencies on the relative abundance of bacterial plant pathogens was not as significant (Figure 1B).

### 3.2 The effect of stover mulching on the composition of soil microbial community

#### 3.2.1 Effects on bacterial community

**Figure 2:**
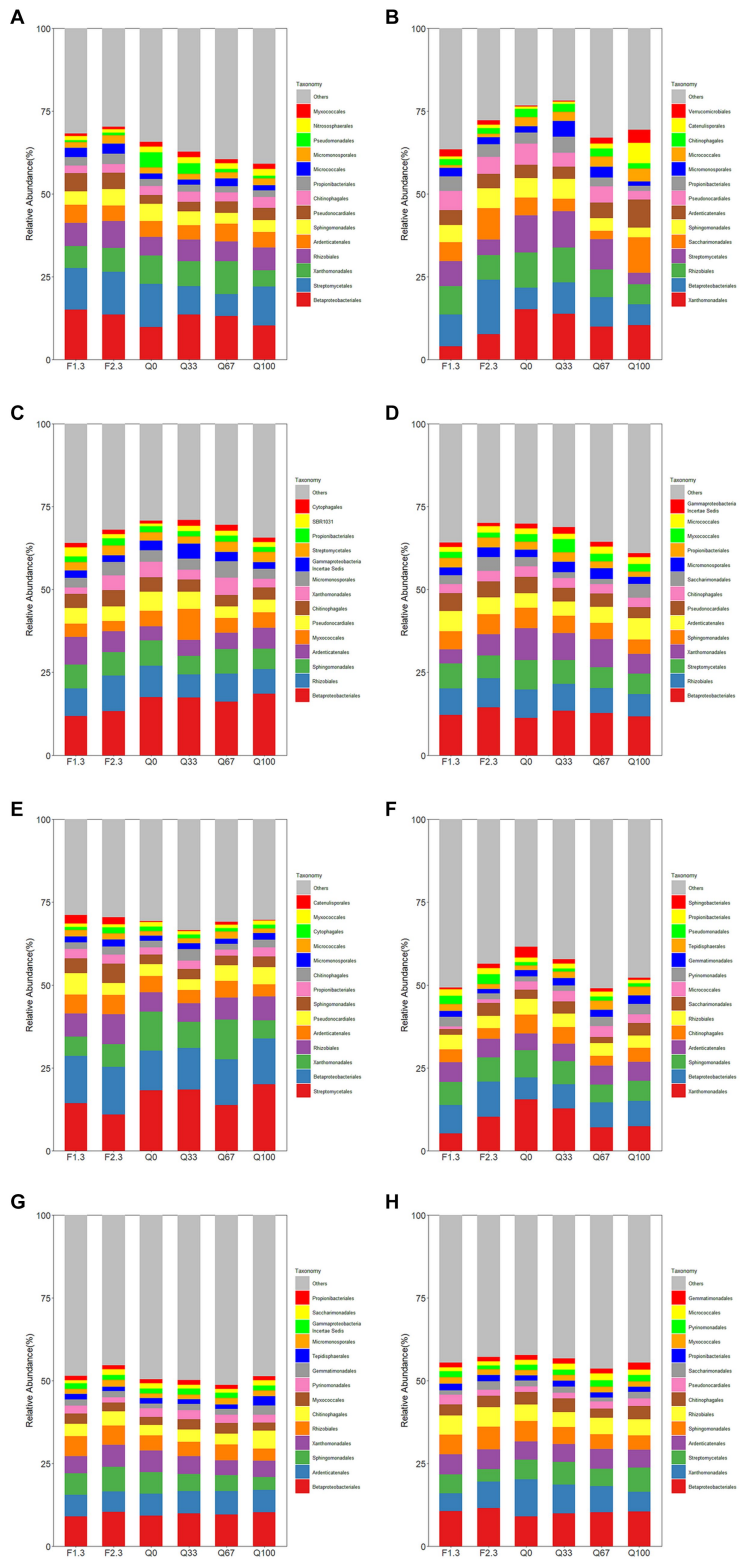
Relative abundance of root endosphere **(A–D)** and rhizosphere **(E–H)** bacteria at the level of the order of magnitude at the stage of jointing **(A,E)**, at the stage of flowering **(B,F)**, at the stage of mature **(C,G)**, and throughout the growth period **(D,H)**.

In the rhizosphere, both F1/3 and F2/3 increased the relative abundance of Rhizobiales totally period compared to Q0 (Figure 2H). And in the rhizosphere, F1/3 also demonstrated an elevated relative abundance of Rhizobiales throughout their entire growth period compared to Q0 (Figure 2H). Within the endosphere, during the jointing stage, all experimental groups showed a slight increase in the relative abundance of Rhizobiales compared to Q0, with F2/3 demonstrating the most pronounced enhancement (Figure 2A). On the mature stage, only F2/3 exhibited a higher relative abundance of Rhizobiales compared to Q0 (Figure 2C). In the rhizosphere, the laws for the jointing and flowering stages were consistent with that observed in the endosphere, with differences emerging only at the mature stage. During this stage, an apparent promotional effect on the relative abundance of Rhizobiales was seen in F1/3 and F2/3 (Figure 2G).

Overall, the relative abundance of Pseudomonadales was not notably high (Figure 2). However, it showed distinct distribution patterns within the endosphere during the jointing stage and in the rhizosphere during the flowering stage. During the flowering stage, within the rhizosphere, F1/3 and F2/3 displayed a higher relative abundance of Pseudomonadales compared to Q0 control (Figure 2F).

#### 3.2.2 Effects on fungal community

**Figure 3:**
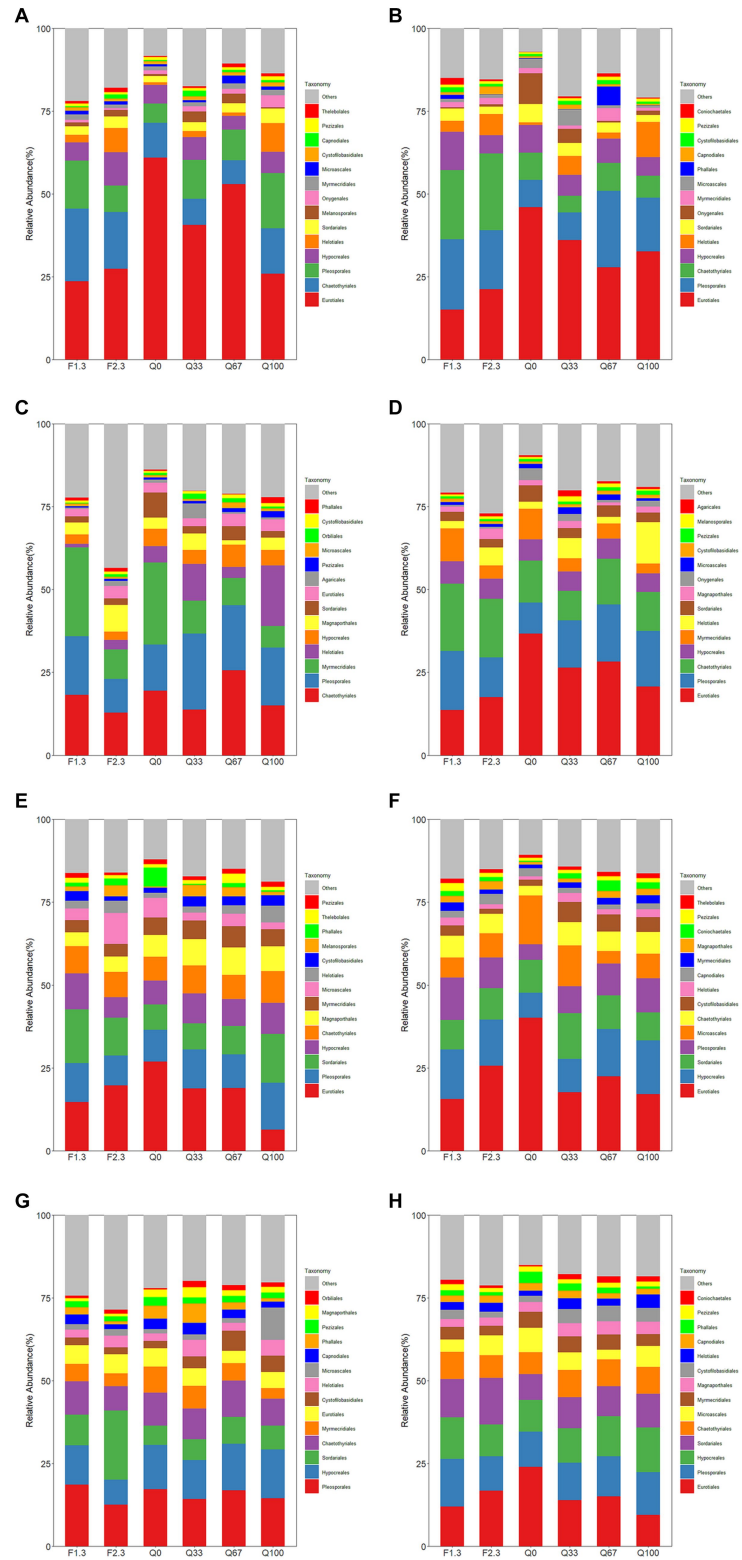
Relative abundance of root endosphere **(A–D)** and rhizosphere **(E–H)** fungi at the level of the order of magnitude at the stage of jointing **(A,E)**, at the stage of flowering **(B,F)**, at the stage of mature **(C,G)**, and throughout the growth period **(D,H)**.

Overall, the total relative abundance of Hypocreales in the endosphere was not significantly affected by different types of stover mulching to the field (Figure 3D). However, at different growth stages, distinct mulches were observed due to these various practices. Relative to Q0, during the jointing stage, F2/3 notably promoted the relative abundance of Hypocreales (Figure 3A). At the flowering stage, only F1/3 increased the relative abundance of Hypocreales; conversely In the rhizosphere, throughout the entire growth period, the relative abundance of Hypocreales was higher in all experimental groups than in Q0 (Figure 3H). Among them, F1/3, Q67, and Q100 had more evident promoting effects on the relative abundance of Hypocreales. During the jointing and flowering stages, almost all experimental groups showed varying degrees of increase in the relative abundance of Hypocreales as compared to Q0, with the highest increments observed in the F1/3 and Q100 (Figures 3E,F).

During the jointing and flowering stages, the relative abundance of Eurotiales in Q0 was highest among all groups for both the endosphere and rhizosphere (Figure 3). And by the mature stage, the relative abundance of Eurotiales was notably diminished (Figures 3C,G). In the endosphere, F1/3 exerted the most suppressive effect on the relative abundance of Eurotiales, maintaining the lowest relative abundance among all groups from the jointing stage through to the mature stage (Figures 3A–D).

### 3.3 The relationship between microbial communities and soil carbon and nitrogen

#### 3.3.1 Bacterial community

**Figure 4:**
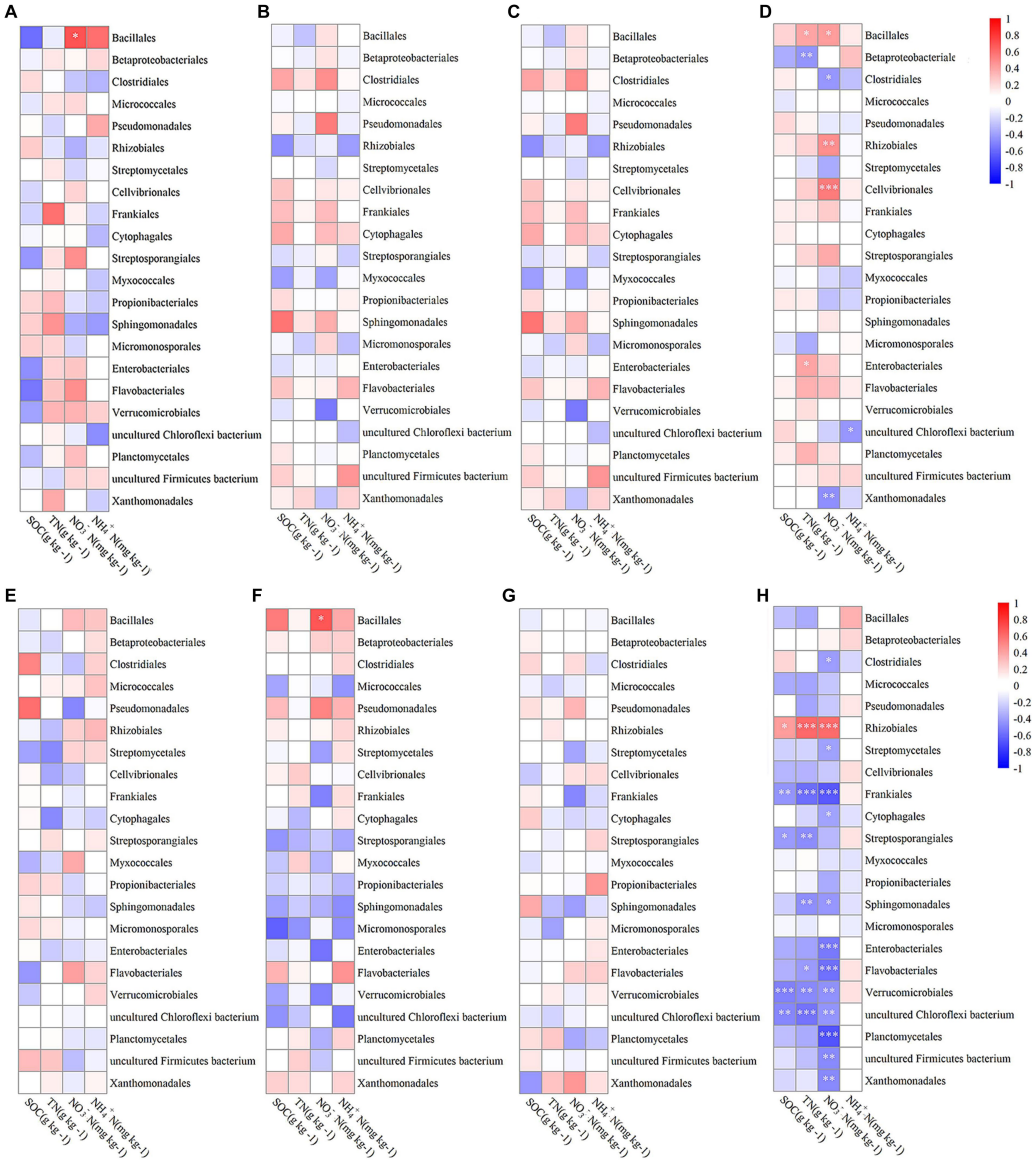
The Spearman correlation coefficients between bacterial communities at the order level in the endosphere **(A–D)** and rhizosphere **(E–H)** across different growth stages: jointing stage **(A,E)**, flowering stage **(B,F)**, mature stage **(C,G)**, and throughout the entire growth period **(D,H)** with soil organic carbon (SOC), total nitrogen (TN), nitrate nitrogen (NO_3_^−^N), and ammonium nitrogen (NH^4+^-N). Statistical significance is denoted as follows: ^*^p < 0.05; ^**^p < 0.01; ^***^p < 0.001.

Figure 4 visually depicted the correlations between various bacterial orders in different growth stages and soil components including SOC, TN, NO_3_^−^N, and NH_4_^+^N. During the jointing stage, Bacillales within the endosphere showed a significant positive correlation with soil NO_3_^−^N levels (*p* < 0.05; Figure 4A). At the flowering stage, Bacillales in the rhizosphere exhibited a similarly significant positive correlation with soil NO_3_^−^N (p < 0.05; Figure 4F). In the mature stage, there were no significant correlations observed between any bacterial orders and the four measured soil properties (Figures 4C,G). Considering the entire growth period, Bacillales within the endosphere demonstrated a significant positive correlation with both soil NO_3_^−^N and NH4 + -N concentrations (p < 0.05; Figure 4D). Furthermore, Rhizobiales within the endosphere had a highly significant positive correlation with soil NO_3_^−^N (*p* < 0.01; Figure 4D), while those in the rhizosphere showed a significant positive correlation with soil SOC (*p* < 0.05; Figure 4H) and an extremely significant positive correlation with both NO_3_^−^N and NH_4_^+^N (*p* < 0.001; Figure 4H).

#### 3.3.2 Fungal community

**Figure 5:**
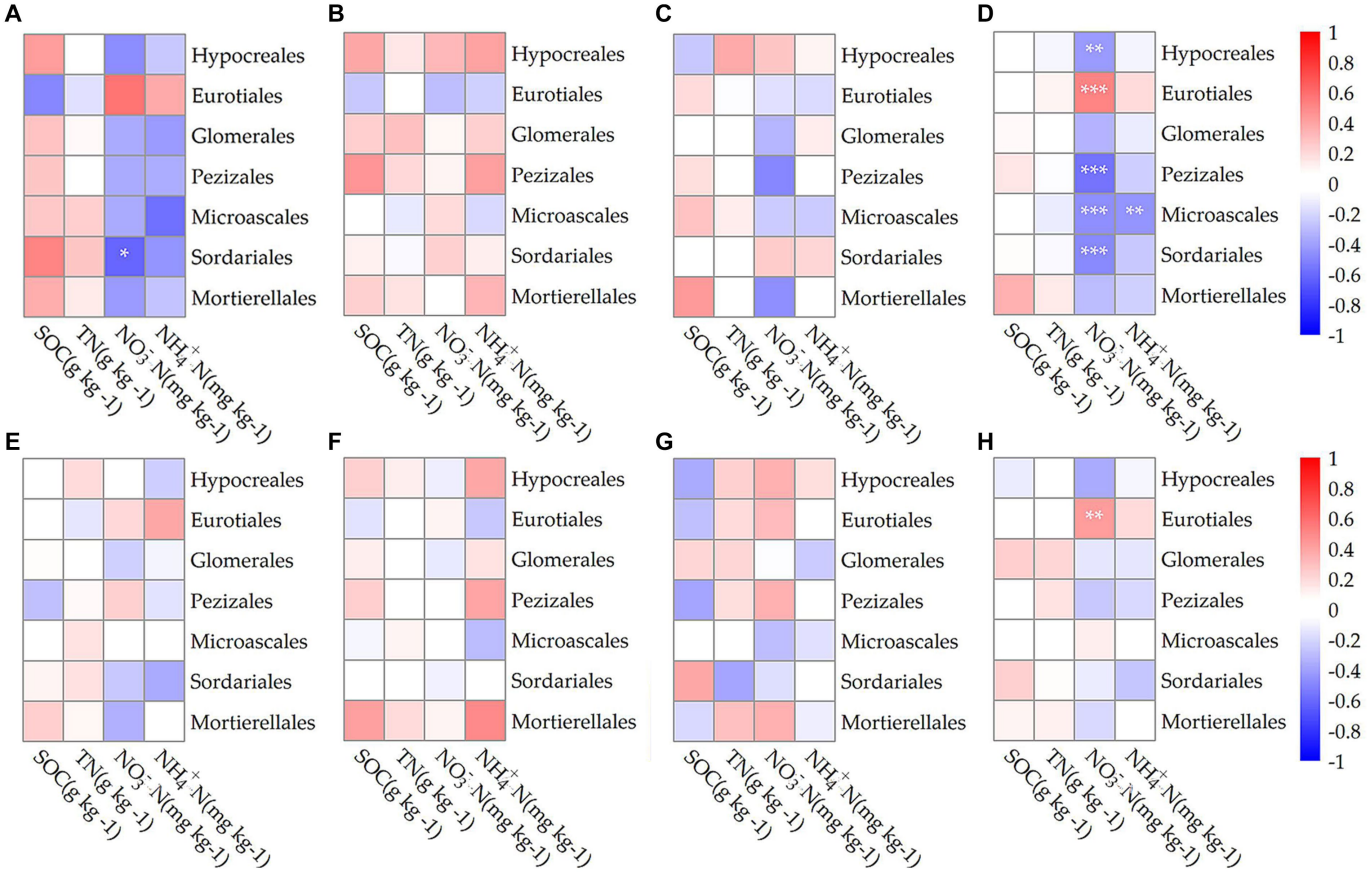
The Spearman correlation coefficients between fungi communities at the order level in the endosphere **(A–D)** and rhizosphere **(E–H)** across different growth stages: jointing stage **(A,E)**, flowering stage **(B,F)**, mature stage **(C,G)**, and throughout the entire growth period **(D,H)** with soil organic carbon (SOC), total nitrogen (TN), nitrate nitrogen (NO_3_^−^N), and ammonium nitrogen (NH_4_^+^N). Statistical significance is denoted as follows: ^*^p < 0.05; ^**^p < 0.01; ^***^p < 0.001.

Figure 5 illustrated the correlations between various fungal orders at different growth stages and soil properties, including SOC, TN, NO_3_^−^N, and NH_4_^+^N. Overall, in the endosphere, Hypocreales showed a significant negative correlation with soil NO_3_^−^N levels (*p* < 0.01; Figure 5D), while Eurotiales exhibited an extremely significant positive correlation with soil NO_3_^−^N (*p* < 0.001; Figure 5D). In the rhizosphere, Eurotiales had a significant positive correlation with soil NO_3_^−^N (*p* < 0.01; Figure 5H). Regarding the Penicillium genus, endophytic Penicillium had a significant positive correlation with soil NO_3_^−^N during the jointing stage (*p* < 0.05) and a significant negative correlation with soil SOC during the flowering stage (*p* < 0.05). Considering the entire growth period, endospheric Penicillium demonstrated an extremely significant positive correlation with soil NO_3_^−^N (*p* < 0.001), while rhizospheric Penicillium showed a significant positive correlation with soil NO_3_^−^N (*p* < 0.01).

### 3.4 Effects of stover mulching quantity and frequency on microbial community structure

#### 3.4.1 Effects on bacterial structure

**Figure 6:**
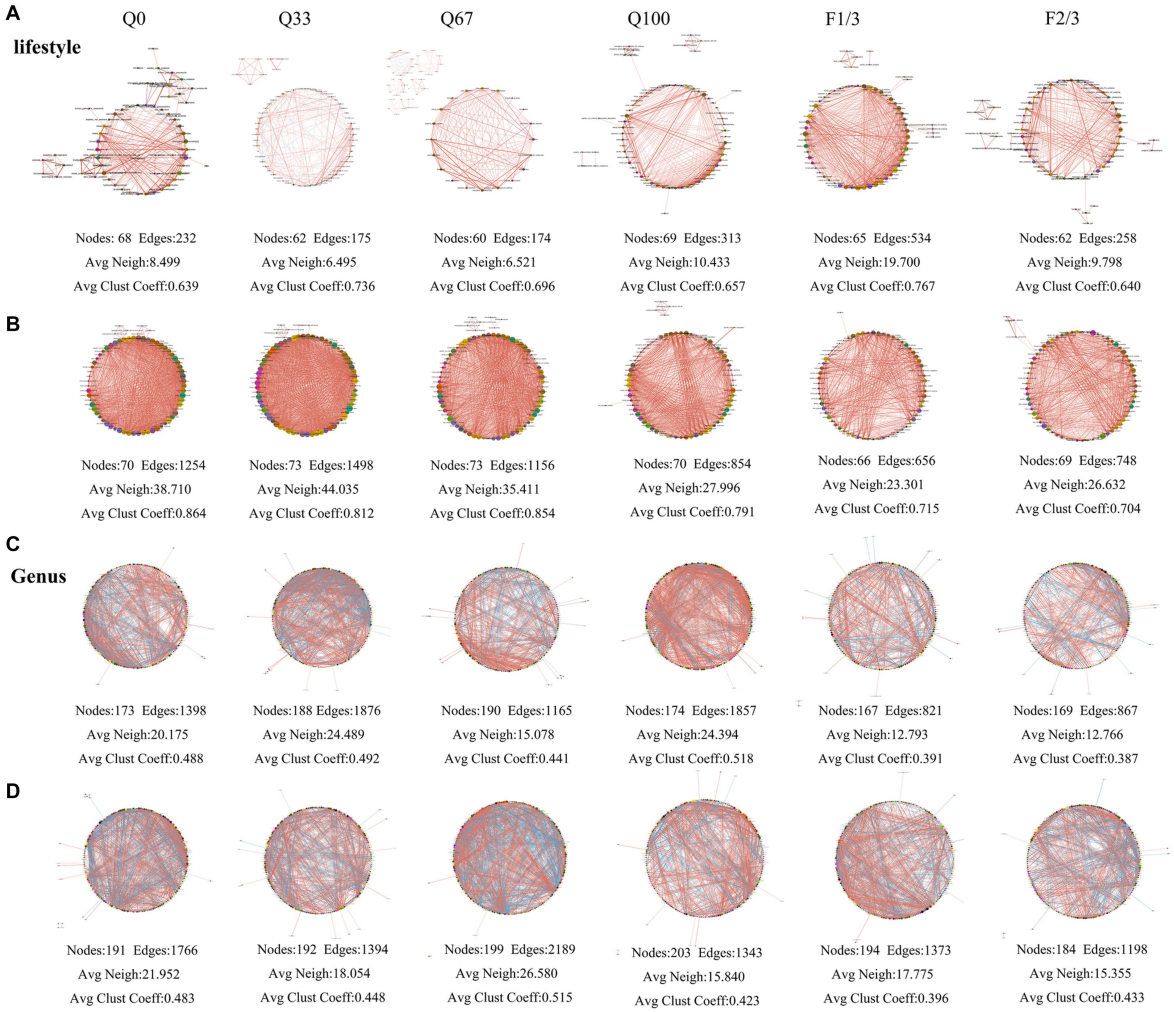
The bacterial community networks at the level of lifestyle **(A,B)** and genus **(C,D)** in the endosphere **(A,C)** and rhizosphere **(B,D)** under varying quantities and frequencies of mulches.

At different stover return quantity and frequency, the topological structures of bacterial community networks exhibited distinct differences (Figure 6). At the lifestyle level, the bacterial community network in the endosphere under Q100 treatment comprised 69 nodes connected by 313 edges, and the average number of neighbors in the network under Q100 treatment was higher than that of the other three networks, indicating a closer relationship among bacterial taxa (Figure 6A). Under F1/3 treatment, the endosphere community network comprised 65 nodes and 534 edges, with both its average number of neighbors and average clustering coefficient significantly higher than that of the network in the F2/3 treatment (Figure 6A). The rhizosphere bacterial community network under Q33 treatment consisted of 73 nodes and 1,498 edges, featuring a higher average number of neighbors compared to the networks under other treatments. The rhizosphere bacterial network in the F2/3 treatment group consisted of 69 nodes with 748 edges, and having a higher average number of neighbors than that of the F1/3 treatment (Figure 6B).

At the genus level, the endosphere bacterial community network under Q0 treatment consisted of 173 nodes and 1,398 edges, while it had 190 nodes and 1,165 edges in Q67 treatment, 174 nodes and 1,857 edges in Q100 treatment, and under Q33 treatment, the network featured 188 nodes connected by 1,876 edges with an average number of neighbors higher than the other three networks, indicating a more complex network structure (Figure 6C). In F1/3 treatment, the network comprised 167 nodes and 821 edges, and for F2/3 treatment, there were 169 nodes and 867 edges (Figure 6C). The rhizosphere bacterial network under Q0 treatment consisted of 191 nodes and 1,766 edges. Under Q33 treatment, it had 192 nodes connected by 1,394 edges. The network for Q67 treatment comprised 199 nodes with 2,189 edges, and in Q100 treatment, it featured 203 nodes and 1,343 edges. Obviously, both the average number of neighbors and the average clustering coefficient in Q67 network were higher than those in the other three treatments (Figure 6D). The network for the F1/3 group consisted of 194 nodes and 1,373 edges, indicating better associations among bacterial genera compared to the F2/3, which had 184 nodes and 1,198 edges. And the network for the F1/3 group also featured a higher average number of neighbors (Figure 6D).

#### 3.4.2 Effects on fungal community structure

**Figure 7:**
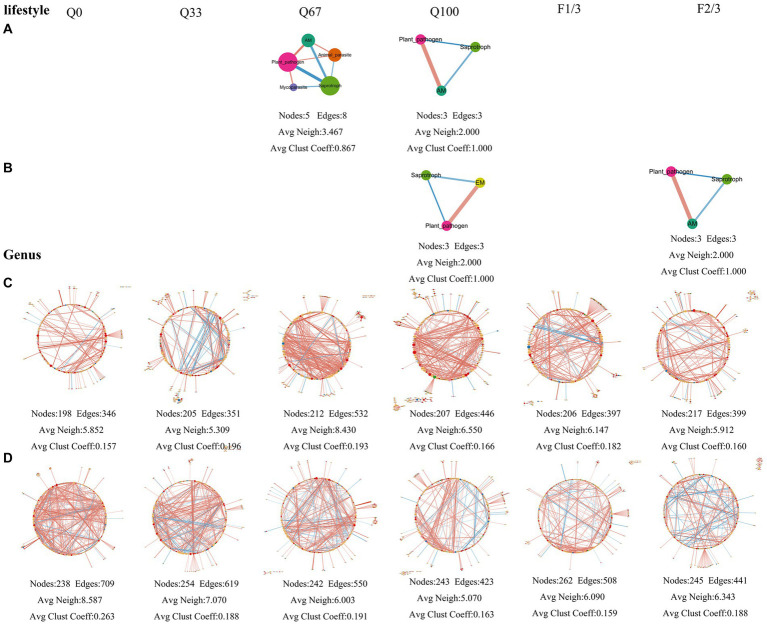
The fungi community networks at the level of lifestyle **(A,B)** and genus **(C,D)** in the endosphere **(A,C)** and rhizosphere **(B,D)** under varying quantities and frequencies of mulches.

Under different quantity and frequency of stover mulching, there were also significant differences in the topology of fungal networks (Figure 7). At the genus level, the endosphere fungal community network suggested that a stover return quantity of Q67 result in stronger associations among different genera (Figure 7C). Regarding the rhizosphere fungal community network, under Q33 treatment, it was composed of 238 nodes and 709 edges, with both its average number of neighbors and average clustering coefficient higher than the other three treatments, indicating that Q0 enhanced the associations among fungal genera (Figure 7D). The network had better inter-genera associations when the straw return frequency was at F1/3 (Figure 7D). The rhizosphere fungal community network consisted of 238 nodes and 709 edges in Q0 treatment, and the network had a higher average number of neighbors and average clustering coefficient compared to the other three treatments, suggesting that Q0 enhanced the associations among fungal genera (Figure 7D). The stover mulching frequency of F1/3 led to stronger inter-genera associations among fungi (Figure 7D).

## 4 Discussion

### 4.1 Frequency and quantity of stover mulching affected the composition and structure of microbial community

Compared to the control group, the bacterial community structures within the rhizosphere and endosphere in different experimental groups were relatively similar, while the fungal community structures exhibited greater variations. Research by Wakelin et al. (2007) suggests that the impact of stover mulching on fungal community structures in the soil is more pronounced than its impact on bacterial community structures. This phenomenon was still applicable both within the endosphere and rhizosphere. Additionally, within the fungal community, the influence of stover mulching on the endosphere was stronger than its impact on the rhizosphere, whereas no such trend was observed in bacterial communities. This may be explained by the morphology of fungi in the soil. Fungi can form a mycelial network with plant roots, facilitating the connection between roots and soil. Hence, they are more susceptible to soil environment changes compared to bacterial communities in the endosphere and rhizosphere (Song et al., 2023). The microbial communities within the endosphere and rhizosphere can be categorized into two systems based on their ecological niches. The endophytic microbial community in the endosphere often engages in symbiosis with plants, while the rhizospheric microbial community serves as a bridge between plant roots and the soil (Song et al., 2023). The fungal community in the endosphere relies more on the mycelial network; therefore, changes in the mycelial network result in more noticeable variations in the fungal community of the endosphere.

Some studies suggest that a high-frequency while small-quantity mulch is more beneficial for soil microbial communities (Kou et al., 2020; Yang et al., 2022). However, in our current study, the enrichment of Rhizobiales in the conditions of high-frequency mulch did not exist both in the endosphere and in the rhizosphere. Although the high-frequency while small-quantity mulch benefits the soil microbial community, it may not necessarily favor the microbial communities within the endosphere and rhizosphere. Studies have shown that certain members of the Rhizobiales order exhibit nitrogen-fixing capabilities and can form stable symbiotic relationships with plants, stimulating plant growth and enhancing productivity (Yang et al., 2022). The observation that F1/3 and F2/3 increase the relative abundance of Rhizobiales in both the endosphere and rhizosphere suggests that F1/3 and F2/3 may be beneficial to the composition of endophytic and rhizosphere microbial communities. And we found that stover mulching effectively inhibited the relative abundance of bacterial plant pathogen, while F1/3 had the most obvious inhibition among experimental groups. Similarly, literature indicates that F1/3 and F2/3 exhibit the lowest abundance of nitrifying bacteria and anaerobic nitrifying bacteria, the highest abundance of nitrogen-fixing bacteria, and F2/3 significantly reduces the relative abundance of fungi plant pathogens in the rhizosphere (Song et al., 2022). Multiple lines of evidences suggest that frequency but not the quantity of stover mulching plays a dominant role in influencing the microbial communities within the endosphere and rhizosphere. Moreover, the low-frequency while high-intensity mulch leads to a healthier microbial composition in the endosphere and rhizosphere.

Some research has revealed that the quantity of stover mulching is a crucial factor influencing the correlation of soil microbial communities. In comparison to other levels of stover mulching, a moderate level of stover mulching leads to a more complex network of soil microbial community, while a small quantity of stover mulching (less than 50% mulching) reduces the complexity of the network (Wang et al., 2021). In our current study, we found that Q33 reduced the complexity of the rhizospheric bacterial genus-level community network, similar to the trends observed in soil microbial networks (Wang et al., 2021). However, the bacterial community network at the genus level within the endosphere was found to be most complex under the stover mulching quantity of Q33, while Q67 significantly reduced the interconnectivity among communities, even resulting in lower association levels than those observed in the Q0 group. This could be attributed to the differences between endophytic and rhizospheric microorganisms, where plants exhibit a stronger selectivity for endophytic microorganisms (Hardoim et al., 2008; Berendsen et al., 2012). Unlike rhizospheric microorganisms, endophytic microorganisms experience fewer biotic and abiotic stresses within plant tissues, and their abundance is less influenced by soil nutrient levels (Wang et al., 2016; Chen et al., 2020). Furthermore, this explains why, in comparison to rhizospheric microorganisms, the impact of stover mulching frequency on the correlation of endophytic microorganisms at the genus level is not as significant. Our study indicates that microbial community correlations vary significantly under different quantities and frequencies of stover mulching, and there is substantial dissimilarity between endophytic and rhizospheric microorganisms. In this study, the stover mulching quantities that resulted in the most complex bacterial community networks at the genus level for endophytic and rhizospheric microorganisms were Q33 and Q67, respectively. More complex community networks may signify stronger interactions among microorganisms, allowing more microorganisms to share ecological niches (Berry and Widder, 2014), thereby promoting the improvement of plant endophytic or rhizospheric environments.

### 4.2 Relationship between microbial communities and soil carbon and nitrogen

Many microorganisms have a significant impact on plant growth and soil physicochemical properties. Under natural conditions, Rhizobiales generally do not form nodules with non-leguminous plants but can colonize the endosphere or rhizosphere such as rice and corn, acting as Plant Growth-Promoting Rhizobacteria (PGPR) to enhance plant growth (Baset Mia and Shamsuddin, 2010). Therefore, the relative abundance of Rhizobiales in the rhizosphere was highly significant correlated with soil NO^3−^N and NH^4+^N levels. Previous research has shown that many groups within Bacillales can fix atmospheric nitrogen, explaining the significant positive correlation observed in this study between the relative abundance of endophytic and rhizospheric Bacillales and soil NO^3−^N levels (Ding et al., 2005; Yousuf et al., 2017). Bacillales have been reported to be used as Plant Growth-Promoting Rhizobacteria (PGPR) in the cultivation of various crops and horticultural plants, contributing to the solubilization of mineral elements. Many species within Bacillales, such as *Bacillus subtilis* and Bacillus amyloliquofaciens, can produce various antibiotics. They also support plant growth by producing plant hormones, releasing ammonia from nitrogen-containing organic compounds, and increasing the plant’s demand for nutrients, thereby promoting nitrogen uptake by plants (Goswami et al., 2014). This explains the significant positive correlation observed in this study between the relative abundance of endophytic Bacillales and soil NH^4+^N levels.

Corn stover organic matter undergoes two processes in the soil, mineralization and humification. Mineralization involves the breakdown of organic matter into simple inorganic compounds through microbial action, which is a crucial pathway for Soil Organic Carbon (SOC) loss (Thuriès et al., 2001). Humification is a process that retains nutrients in the soil, and humus is the most stable component of soil organic matter (Adam et al., 1985; Pei et al., 2015). The decomposition of cellulose in corn stover is a vital step in its degradation. Some strains of filamentous fungi from Penicillium exhibit strong secretion capabilities of cellulolytic enzymes, and they have advantages in terms of enzyme performance and strain growth rate (Gusakov, 2011). Research has shown that the relative abundance of endophytic Penicillium is significantly negatively correlated with SOC levels during the flowering stage, which may be related to the secretions produced by endophytic Penicillium. When interacting with plants, these fungi secrete antibiotics and other biocontrol agents that positively influence plant growth, thereby promoting nutrient release in the soil (Bashan and De-Bashan, 2010). Stover mulching can both enhance SOC recovery and improve soil fertility through increasing carbon input (Fan et al., 2014b); however, it may also indirectly decrease SOC content due to the promotion of plant growth by specific fungi like Penicillium. The effect of stover mulching on soil carbon sequestration is dual-sided, and how to regulate the balance between carbon storage and release remains an area for further research.

## 5 Conclusion

The study found that the quality and frequency of stover mulching significantly influence the composition and structure of endophytic and rhizospheric microbial communities. Nitrogen-fixing bacteria and rhizosphere-promoting microorganisms showed significant correlations with SOC, NO_3_^−^N, and NH_4_^+^N. Cellulose-degrading fungi were notably related to SOC content. Different amounts and frequencies of stover mulching had varying impacts on the microbiota within both the endosphere and rhizosphere. The conditions F1/3 and F2/3 proved most beneficial for the composition of endophytic and rhizospheric microbial communities, while Q33 and Q67 were optimal for the structural complexity of endophytic and rhizospheric microbial communities, respectively.

Stover mulching has a dual impact on soil carbon sequestration, and the balance between carbon fixation and release is likely to be a focal point of future research. This study highlights that certain stover mulching conditions that are favorable to overall soil microbiota may not necessarily be advantageous for the composition of endophytic and rhizospheric microbial communities, and conditions that promote a beneficial composition in these root-associated microbiota might not always favorably influence their structural organization. The underlying reasons for this phenomenon require further exploration. This research holds significant ecological implications for agricultural production and conservation practices.

## Data availability statement

The datasets presented in this study can be found in online repositories. The names of the repository/repositories and accession number(s) can be found in the article/Supplementary material.

## Author contributions

HL: Data curation, Formal Analysis, Investigation, Writing – original draft. YZ: Data curation, Formal Analysis, Investigation, Writing – original draft. WS: Conceptualization, Methodology, Project administration, Writing – review & editing. JX: Data curation, Formal Analysis, Writing – review & editing. JG: Funding acquisition, Project administration, Resources, Writing – review & editing.
