# Supplementary figures and images for: Effects of frequency and amount of stover mulching on the microbial community composition and structure in the endosphere and rhizosphere
Source: Front Microbiol. 2024 Feb 26;15:1372471. doi: 10.3389/fmicb.2024.1372471 (PMC10925793; doi:10.3389/fmicb.2024.1372471)

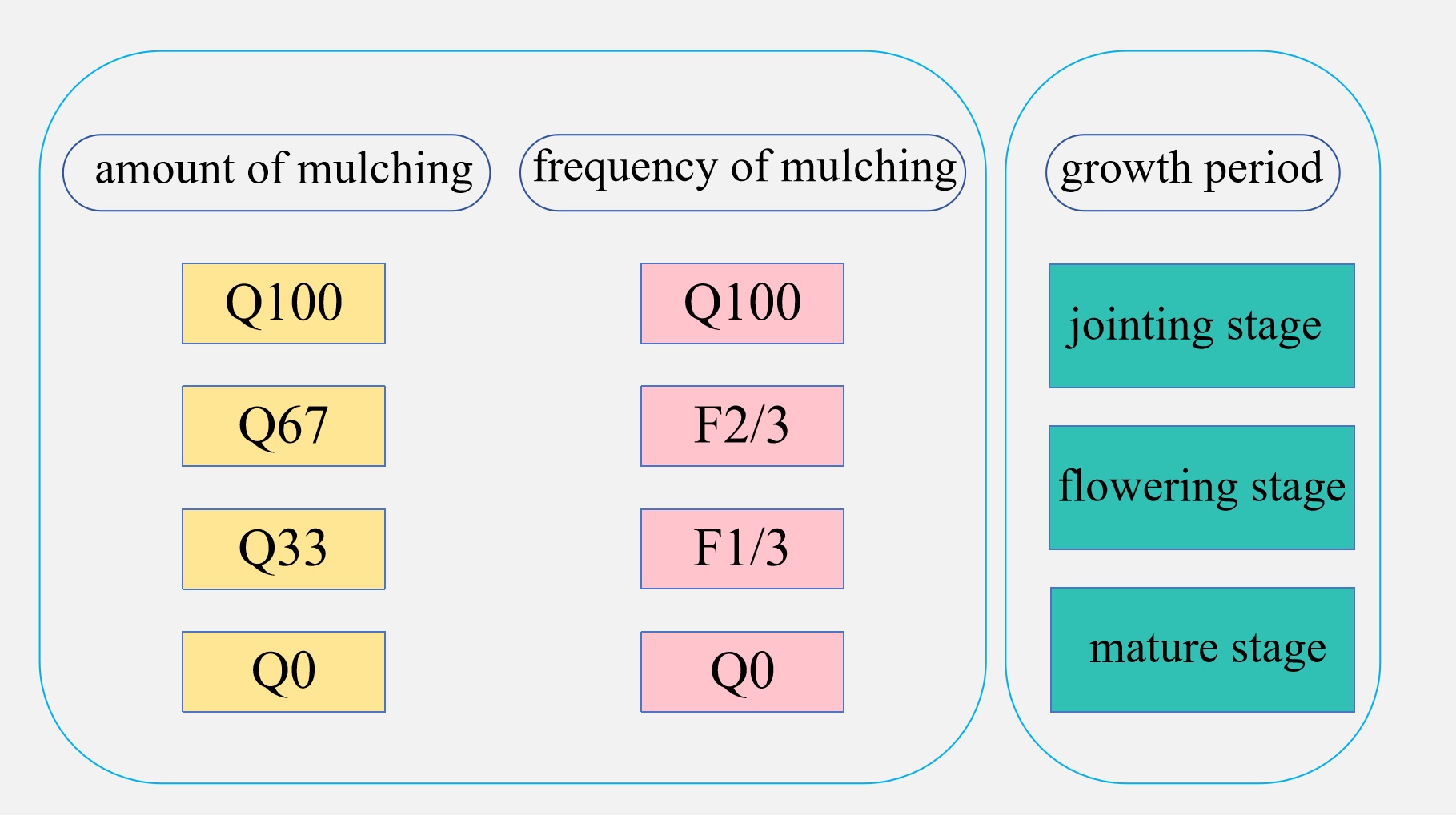

Supplement: Supplementary file 1 [file Image_1.JPEG]
